# Regulation of Rubisco activity by interaction with chloroplast metabolites

**DOI:** 10.1042/BCJ20240209

**Published:** 2024-08-05

**Authors:** Ana K.M. Lobo, Douglas J. Orr, Elizabete Carmo-Silva

**Affiliations:** Lancaster Environment Centre, Lancaster University, Lancaster LA1 4YQ, U.K.

**Keywords:** docking analysis, *Oryza sativa*, Rubisco inhibition, Rubisco regulation, stroma metabolites

## Abstract

Rubisco activity is highly regulated and frequently limits carbon assimilation in crop plants. In the chloroplast, various metabolites can inhibit or modulate Rubisco activity by binding to its catalytic or allosteric sites, but this regulation is complex and still poorly understood. Using rice Rubisco, we characterised the impact of various chloroplast metabolites which could interact with Rubisco and modulate its activity, including photorespiratory intermediates, carbohydrates, amino acids; as well as specific sugar-phosphates known to inhibit Rubisco activity - CABP (2-carboxy-d-arabinitol 1,5-bisphosphate) and CA1P (2-carboxy-d-arabinitol 1-phosphate) through *in vitro* enzymatic assays and molecular docking analysis. Most metabolites did not directly affect Rubisco *in vitro* activity under both saturating and limiting concentrations of Rubisco substrates, CO_2_ and RuBP (ribulose-1,5-bisphosphate). As expected, Rubisco activity was strongly inhibited in the presence of CABP and CA1P. High physiologically relevant concentrations of the carboxylation product 3-PGA (3-phosphoglyceric acid) decreased Rubisco activity by up to 30%. High concentrations of the photosynthetically derived hexose phosphates fructose 6-phosphate (F6P) and glucose 6-phosphate (G6P) slightly reduced Rubisco activity under limiting CO_2_ and RuBP concentrations. Biochemical measurements of the apparent *V*_max_ and *K_m_* for CO_2_ and RuBP (at atmospheric O_2_ concentration) and docking interactions analysis suggest that CABP/CA1P and 3-PGA inhibit Rubisco activity by binding tightly and loosely, respectively, to its catalytic sites (i.e. competing with the substrate RuBP). These findings will aid the design and biochemical modelling of new strategies to improve the regulation of Rubisco activity and enhance the efficiency and sustainability of carbon assimilation in rice.

## Introduction

Photosynthesis is the primary process that supports life on Earth. Through a series of complex reactions triggered by light, oxygen and sugars are produced from water and inorganic CO_2_, respectively. The first step in the assimilation of CO_2_ is catalysed by Rubisco (ribulose-1,5-bisphosphate carboxylase/oxygenase), a large (∼550 kDa), complex, and highly regulated enzyme [1]. To counteract its complexity, plants accumulate large amounts of Rubisco (30–50% of total soluble protein in C3 plants) [2]. However, carboxylation by Rubisco is often still a limiting step, and a key target to improve photosynthesis and crop yield in a world with a changing climate and an increasing population [3,4]. Rubisco has relatively slow catalysis (∼2–5 CO_2_ fixed per second), catalyses two competing reactions (carboxylation and oxygenation), and its activity is highly regulated by the chloroplast stroma environment [5]. Both the external environment surrounding the leaf and the consequent internal environment in the chloroplast stroma, surrounding Rubisco, contribute to regulating Rubisco activity and determining CO_2_ assimilation rates.

To be catalytically competent, Rubisco needs to be carbamylated by the binding of an ‘activator CO_2_’ and a ‘stabilizer’ Mg^2+^ to a specific Lysine (Lys_201_) in its catalytic site [6]. However, both the carbamylated or uncarbamylated form of Rubisco can become locked in an inactive conformation by the binding of inhibitory sugar phosphate derivatives (which are structurally similar to the substrate RuBP (ribulose-1,5-bisphosphate), Supplementary Table S1). Rubisco activity is restored by the co-ordinated reaction of its chaperone Rubisco activase (Rca) and specific phosphatases which release and degrade these inhibitors, respectively, from Rubisco catalytic sites (detailed in [7]). The natural inhibitor, CA1P (2-carboxy-d-arabinitol 1-phosphate), is synthesised in some species during low light or dark conditions by yet-unknown pathways and degraded by CA1Pase (CA1P phosphatase) upon light activation of photosynthesis. Other inhibitory sugar phosphates, e.g. XuBP (xylulose-1,5-bisphosphate) and PDBP (d-glycero-2,3-pentodiulose 1,5-bisphosphate), are produced by catalytic misfires during Rubisco carboxylation or oxygenation activities. The production of these misfire products and how they affect Rubisco activity are not as well documented but are likely affected by the enzyme kinetic traits and conformation, which are dependent on the surrounding environment and the species [8,9].

The concentrations of several chloroplast metabolites change in response to light. For instance, upon a low-to-high light transition 6-phosphogluconate decreases and NADPH increases in the stroma; and both act as positive effectors that interact with Mg^2+^ to maintain Rubisco activity at low or steady-state light conditions, respectively [10–12]. Under steady-state light conditions, the concentration of metabolites produced downstream of Rubisco carboxylation or oxygenation activity increases. Some *in vitro* studies have been performed over the last five decades to understand how different chloroplast metabolites affect Rubisco activity [10,13–15]. However, the findings are fragmented and in some cases contradictory, as some studies used crude leaf extracts while others used purified Rubisco enzyme, and many studies used metabolite concentrations that are unlikely to be physiologically relevant.

3-phosphoglycerate (3-PGA) is the product of Rubisco carboxylation while 3-PGA and 2-phosphoglycolate (2-PG) are the products of Rubisco oxygenation reactions (detailed in [1]). The first (3-PGA) is a triose-phosphate used for synthesis of sugars and other molecules in plant metabolism, including amino acids. The second (2-PG) is considered metabolic waste, toxic, and needs to be regenerated to 3-PGA through the photorespiratory pathway. It has been hypothesized that the accumulation of photosynthesis-derived sugars and photorespiratory metabolites decreases photosynthesis and plant growth by down-regulating Rubisco activity [16–19]. However, studies testing the direct effect of individual metabolites on Rubisco *in vitro* activity are scarce. In a study focusing on sugars (at physiological concentration ranges), spinach Rubisco activity was not affected by fructose 6-phosphate (F6P), and was little affected by fructose-1,6-bisphosphate (FBP) and 3-phosphoglycerate (3-PGA) [13]. The photorespiratory metabolite glyoxylate decreased Rubisco activity when tested with isolated intact spinach chloroplasts, but not with purified Rubisco [14,15]. The authors suggested that Rubisco activity is indirectly affected by glyoxylate via down-regulation of Rca, without a direct interaction with Rubisco. The same authors showed that the amino acids glycine (Gly) and glutamate (Glu) and the respiratory product 2-oxoglutarate did not change Rubisco activity in isolated intact spinach chloroplasts [14].

The number of chloroplast metabolites tested for their effect on Rubisco activity is limited and there have been contradictory or inconclusive results due to differences in assay conditions (i.e. leaf extract, isolated chloroplast, or purified enzyme), and organism-dependent Rubisco conformation and kinetics. Plant metabolism is complex and responds rapidly to internal and external signals through fine-tuned adjustments in enzymatic activities and metabolite concentrations. To better understand how chloroplast metabolites interact and regulate Rubisco activity this study combined *in vitro* enzymatic assays using purified rice Rubisco, complemented by a molecular docking analysis of crystal structures. We investigated the interaction of carbamylated Rubisco with inhibitory sugar phosphates, carbohydrates, photorespiratory compounds and amino acids. The synthetic inhibitory sugar phosphate CABP (2-carboxy-d-arabinitol-1,5-bisphosphate), which has a similar structure to RuBP and a high affinity to carbamylated Rubisco catalytic sites, was used as a reference for a strong inhibitor of Rubisco activity. The results show that only a few chloroplast metabolites have direct effect on Rubisco activity, both in presence of saturating and limiting substrate conditions. The possible mechanisms of interaction with Rubisco are discussed in the context of improving the regulation of the enzyme in crops in current and future climate conditions.

## Results

### Carbamylated rice Rubisco is selectively down-regulated by inhibitory sugar phosphates and 3-PGA

**Figure 1.**
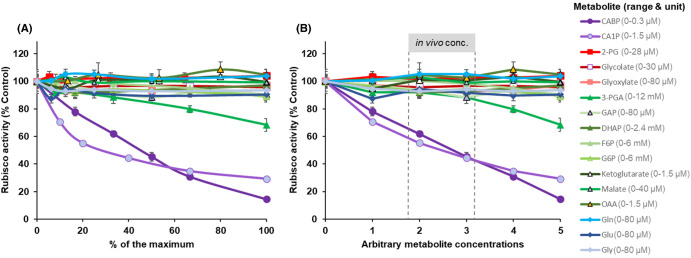
Effect of chloroplast metabolites dose-response curve on rice Rubisco activity. Metabolites concentration expressed as % of the maximum (**A**) and as arbitrary concentrations (**B**). The dashed lines represent the expected *in vivo* chloroplast concentration under steady-state conditions (Supplementary Table S1). The group of each metabolite is represented by colours and symbols (purple/circle for inhibitory sugar phosphates; red/square for photorespiratory compounds; green/triangle for carbohydrates, and blue/diamond for amino acids). Symbols represent the average (±SE) of 3–7 technical replicates for all metabolites except for malate, oxaloacetate and ketoglutarate that had two technical replicates. Control average = 1.23 ± 0.05 µmol mg^−1^ protein min^−1^.

**Figure 2.**
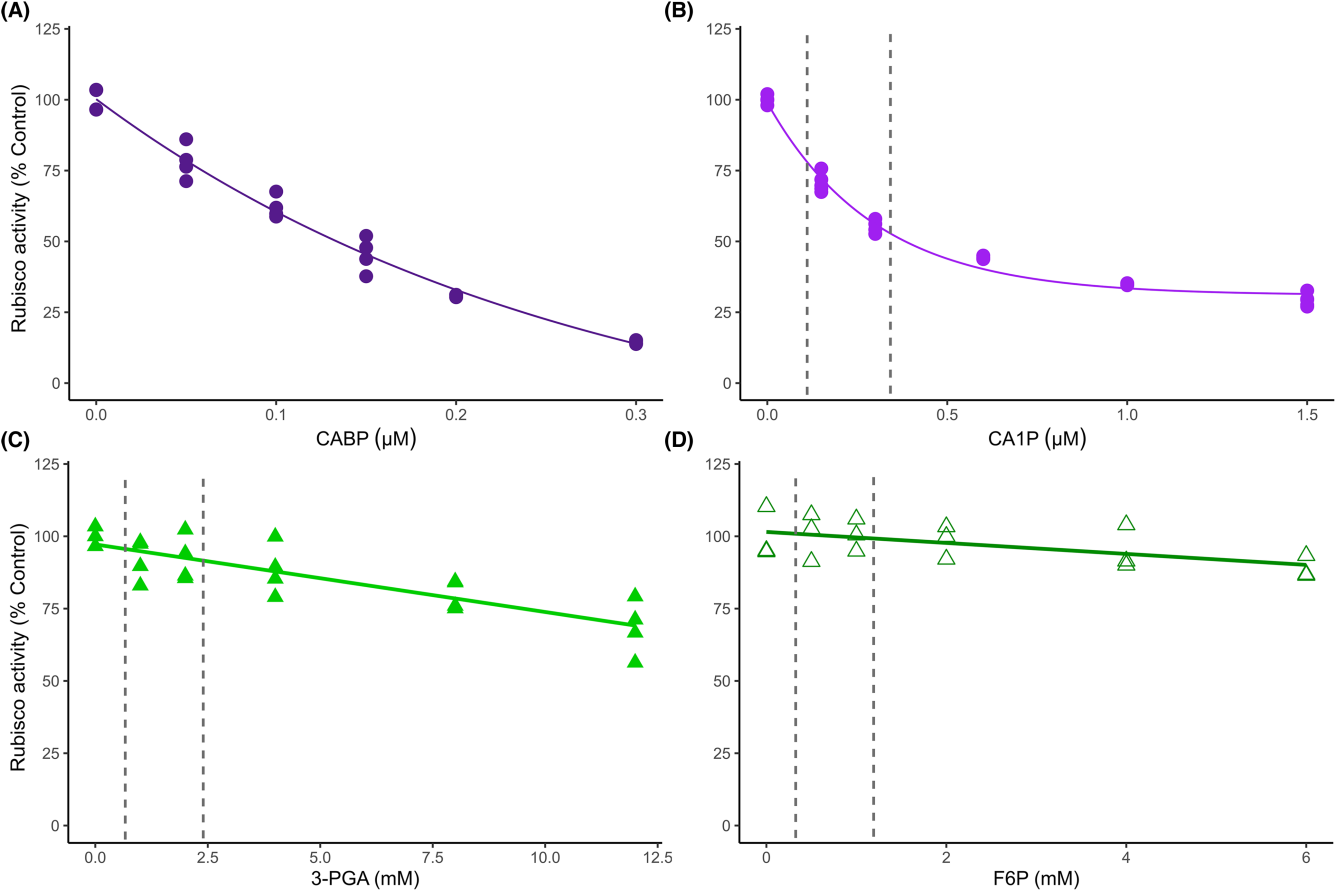
Fitting of chloroplast metabolite dose-response curve on Rubisco activity. The dashed lines represent the expected *in vivo* chloroplast concentrations under steady-state conditions (Supplementary Table S1). Effect of 2-carboxy-d-arabinitol-1,5-bisphosphate (**A**), 2-carboxy-d-arabinitol 1-phosphate (**B**), 3-phosphoglycerate (**C**) and fructose 6-phosphate (**D**) on rice Rubisco activity. Each curve was fitted according to the lowest AIC presented in Supplementary Table S2. Symbols represent individual samples (*n* = 3–5 technical replicates). Control average = 1.23 ± 0.05 µmol mg^−1^ protein min^−1^.

To better understand how carbamylated Rubisco is regulated by chloroplast stroma metabolites, Rubisco *in vitro* activity was tested in the presence of individual metabolites at a range of concentrations. The synthetic inhibitor CABP was used for comparison as it resembles the substrate RuBP (Supplementary Table S1) and has a high affinity to the carbamylated Rubisco catalytic sites. CA1P, a natural Rubisco inhibitor, and other metabolites from the photorespiratory pathway, carbohydrates and amino acids were also tested under saturating substrates and ambient O_2_ concentrations (assay 1, Figure 1). As expected, increasing CABP concentrations linearly decreased Rubisco activity (∼86% at 0.3 µM, *P* < 0.001) when compared with the control (0 metabolite, Figures 1 and 2A, Supplementary Table S2). The maximum concentration of CABP applied (0.3 µM) corresponds to the concentration of Rubisco catalytic sites used. For those metabolites which occur naturally, the range of concentrations used centred on an estimate of expected *in vivo* concentration (Figure 1, Supplementary Table S1). For CA1P, even low concentrations (<0.3 µM) reduced Rubisco activity, with this effect appearing to begin to saturate at higher concentrations, decreasing Rubisco activity by 70% at 1.5 µM (*P* < 0.001, Figures 1 and 2B, Supplementary Table S2). High concentrations of 3-PGA (8 and 12 mM, >2 times of typical *in vivo* concentrations) led to a decrease in Rubisco activity of ∼30% at 12 mM (*P* < 0.001) while the other metabolites tested had little (6 mM F6P decreased Rubisco activity by 11%, *P* < 0.05) or no significant effect on Rubisco activity relative to the control (Figure 1C,D, Supplementary Figure S1 and Table S2). The decrease in Rubisco activity by 3-PGA was not as strong as by CABP or CA1P but presented a strong linear dose-response correlation (*R*^2^ = 0.69) while the linear correlation for reductions in activity due to increasing F6P was weak (*R*^2^ = 0.33, Figure 1, Supplementary Table S2).

### Inhibitory sugar phosphates and 3-PGA compete with RuBP for Rubisco catalytic sites

Apparent Rubisco maximum velocity (*V*_max_) and the substrate affinity (Michaelis-Menten constant — *K_m_*, lower values mean higher affinity) were assessed under limiting concentrations of the substrates CO_2_ (<100 µM) or RuBP (<600 µM), in the presence of atmospheric O_2_ concentrations ($V_{\max}^{CO_{2}}$, $V_{\max}^{\mathrm{RuBP}}$, $K_{m}^{CO_{2}}$ and $K_{m}^{\mathrm{RuBP}}$, Supplementary Figures S2 and S3). In these assays, we focused on the metabolites that affected Rubisco activity under saturating and limiting substrate concentrations (Figure 2, Supplementary Figures S4 and S5). We tested three concentrations of each metabolite: 0 — no metabolite (control), an intermediate concentration, and the highest concentration used in the previous metabolite dose-dependent assays (assay 1, Figure 1). For CABP, the maximum concentration used in this assay was 0.2 µM as at the highest concentration (0.3 µM) Rubisco activity was very low, and for the 3-PGA we used the concentrations that decreased Rubisco activity in assay 1 (8 and 12 mM, Figure 2).

**Figure 3.**
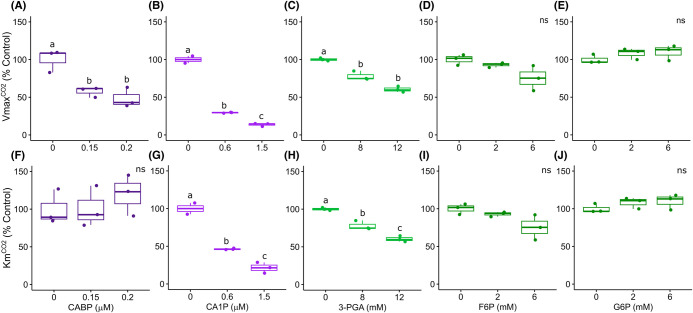
Rubisco kinetics for CO_2_ in the presence of chloroplast metabolites under saturating concentration of RuBP (600 µM). Effect of 2-carboxy-d-arabinitol-1,5-bisphosphate (**A, F**), 2-carboxy-d-arabinitol 1-phosphate (**B, G**), 3-phosphoglycerate (**C, H**), fructose 6-phosphate (**D, I**) and glucose 6-phosphate (**E, J**) on Rubisco maximum velocity — $V_{\max}^{CO_{2}}$ (**A–E**) and Michaelis-Menten constant — $K_{m}^{CO_{2}}$ (**F–J**). Boxes represent the median and first and third quartiles, whiskers represent the range and symbols represent individual samples (*n* = 3 technical replicates, except for 0 µM CA1P with two technical replicates). Different letters represent significant differences at 5% level, according to Tukey's test (*P* ≤ 0.05). ns = not significant. Control average $V_{\max}^{CO_{2}}$ = 2.61 ± 0.2 µmol mg^−1^ protein min^−1^ and $K_{m}^{CO_{2}}$ = 43.49 ± 3.13 µM.

**Figure 4.**
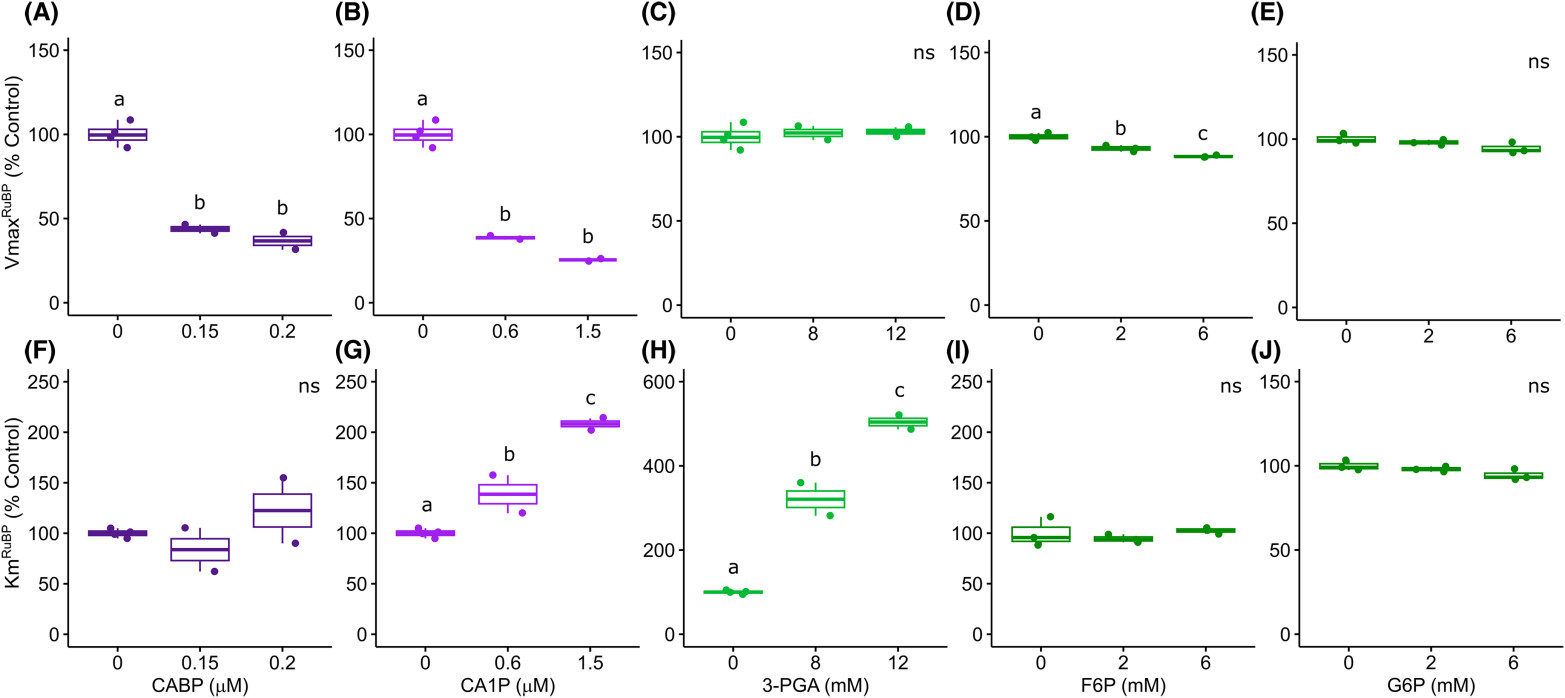
Rubisco kinetics for RuBP in the presence of chloroplast metabolites under saturating concentration of CO_2_ (110 µM). Effect of 2-carboxy-d-arabinitol-1,5-bisphosphate (**A, F**), 2-carboxy-d-arabinitol 1-phosphate (**B, G**), 3-phosphoglycerate (**C, H**), fructose 6-phosphate (**D, I**) and glucose 6-phosphate (**E, J**) on Rubisco maximum velocity — $V_{\max}^{\mathrm{RuBP}}$ (**A–E**) and Michaelis-Menten constant — $K_{m}^{\mathrm{RuBP}}$ (**F–J**). Boxes represent the median and first and third quartiles, whiskers represent the range and symbols represent individual samples (*n* = 2–4 technical replicates). Different letters represent significant differences at 5% level, according to Tukey's test (*P* ≤ 0.05). ns = not significant. Control average $V_{\max}^{\mathrm{RuBP}}$ = 1.59 ± 0.16 µmol mg^−1^ protein min^−1^ and $K_{m}^{\mathrm{RuBP}}$ = 78.08 ± 3.29 µM.

For both CO_2_ and RuBP substrates CABP decreased the apparent *V*_max_ by ∼52% without impacting the apparent *K_m_* (Figure 3A,F). These results suggest that CABP strongly inhibits Rubisco activity by binding very tightly to its catalytic site as reported previously [8,20]. The natural inhibitor, CA1P, decreased both apparent $V_{\max}^{CO_{2}}$ and $V_{\max}^{\mathrm{RuBP}}$ (by 86% and 75%, respectively), while the apparent $K_{m}^{CO_{2}}$ decreased (by 78%) and $K_{m}^{\mathrm{RuBP}}$ was double the control (Figures 3 and 4). This result indicates that CA1P competes with RuBP, but the binding to Rubisco catalytic sites is less tight than CABP. High concentrations of 3-PGA (>8 mM) decreased the apparent $V_{\max}^{CO_{2}}$ and $K_{m}^{CO_{2}}$ (by 35%), while this metabolite did not change the apparent $V_{\max}^{\mathrm{RuBP}}$ and increased the apparent $K_{m}^{\mathrm{RuBP}}$ 5-fold compared with the control (Figures 3 and 4). 3-PGA is an example of classic competitive inhibition where the inhibitor binds more loosely to the catalytic site, competing with RuBP, increasing the apparent $K_{m}^{\mathrm{RuBP}}$ and not affecting the apparent $V_{\max}^{\mathrm{RuBP}}$. Consistent with these observations is that this inhibition decreased at a higher concentration of RuBP. As Rubisco catalyses a reaction with two substrates and requires prior carbamylation of the catalytic site, and because specific sugar-phosphate inhibitors such as CA1P and the synthetic substrate analogue CABP bind tightly to catalytic sites, the determination of the inhibitor constants (*K_i_*) for these metabolites is not straightforward [8,21]. We have used a simplistic method to estimate apparent inhibition constants $\left(K_{i}^{\mathrm{app}}\right)$ that are one order of magnitude lower for CABP and CA1P compared with 3-PGA (Supplementary Table S3).

For most of the metabolites investigated, even at high concentration, there was no significant detectable change in Rubisco activity compared with the control under limiting concentrations of CO_2_ or RuBP (Supplementary Figures S4 and S5). The triose phosphate dihydroxyacetone phosphate (DHAP) slightly increased (13%) Rubisco activity at limiting CO_2_ and the two hexose phosphates F6P and G6P slightly decreased Rubisco activity under limiting concentrations of CO_2_ (by 23% and 11%, respectively) and RuBP (each by 15%). F6P slightly reduced apparent $V_{\max}^{\mathrm{RuBP}}$ and did not change the apparent $V_{\max}^{CO_{2}}$ or the *K_m_* for both substrates, while G6P did not change the apparent *V*_max_ or *K_m_* for both substrates (Figures 3 and 4, Supplementary Figures S2 and S3). High concentrations of F6P and G6P seem to cause a minor decrease in Rubisco activity under limiting substrate concentrations, but the mechanism for this is not clear. The other metabolites tested did not interact directly with carbamylated rice Rubisco in the *in vitro* conditions tested (30°C and pH 8.0).

### Molecular docking analysis

To understand how some of these metabolites may be interacting with Rubisco catalytic sites and causing alterations in catalysis, we carried out molecular docking analyses using the crystallized structure of carbamylated rice Rubisco bound to CABP (PDB ID: 1WDD [12]), and the metabolites that affected Rubisco kinetics in the *in vitro* assays, such as CABP, 3-PGA, F6P and G6P (Figures 3 and 4). Structural studies including the natural Rubisco inhibitory sugar phosphate are scarce, and we are not aware of a published structure with CA1P, hence we used the CABP-bound structure as a close approximation. The PLP.PLP score, which is inversely related to the binding strength, and reflects the sum of interaction contributions (e.g. H-bonds, non-polar, buried and ligand penalty), was lower for CABP, followed by RuBP and 3-PGA, suggesting that, as expected, CABP binds more strongly to Rubisco catalytic site than RuBP or 3-PGA (Table 1). However, the buried contribution (representing the fitting of the ligand in the catalytic site, the lower the better) was lower for RuBP than for other metabolites, indicating a better fitting with lower torsion (ligand penalty, the lower the better) compared with CABP. The PLP.PLP scores determined for the docking analysis with F6P and G6P were relatively low and similar to RuBP and 3-PGA, mainly caused by non-polar contributions, but with higher buried contribution (Table 1). The binding strength of CABP, CA1P and 3-PGA to Rubisco catalytic sites as estimated by the molecular docking analysis corroborates the apparent inhibitor constants $\left(K_{i}^{\mathrm{app}}\right)$ estimated here using a simplistic method for each of these metabolites (Supplementary Table S3).

**Table 1.** Scores from the molecular docking analysis of carbamylated rice Rubisco (PDB ID: 1WDD) interacting with each metabolite (ligand) in arbitrary units.

Ligand	PLP.PLP Score	H-bonds contribution	Non-polar contribution	Buried contribution	Ligand penalty
CABP	−40.89	−13.71	−24.55	−3.33	1.55
RuBP	−35.51	−9.69	−19.08	−6.20	1.31
3-PGA	−22.97	−6.00	−13.59	−1.99	0.26
F6P	−35.65	−9.12	−23.09	−3.18	0.24
G6P	−27.44	−10.59	−20.47	4.71	0.47

CABP, 2-carboxy-d-arabinitol-1,5-bisphosphate; RuBP, ribulose-1,5-bisphosphate; 3-PGA, 3-phosphoglycerate; F6P, fructose 6-phosphate; G6P, glucose 6-phosphate.

**Figure 5.**
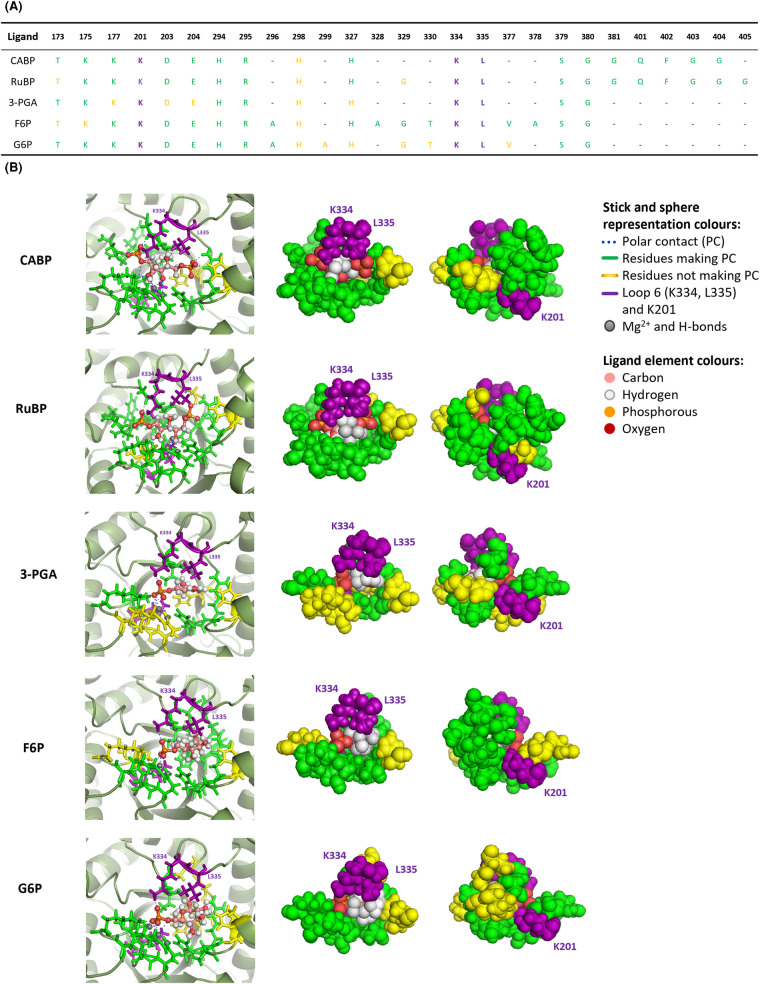
Molecular docking analysis predicted interactions between metabolites and residues around the catalytic site of rice Rubisco. Sequence of amino acids interacting with each ligand in 4 Å distance (**A**) and stick and sphere representation of the best fitting docking analysis (**B**). Bold Lysine 201 and loop 6 (K334, L335) represent polar contact with the ligand.

The sequence of amino acids in the catalytic site that interacts with each metabolite in 4 Å distance is similar for CABP and RuBP, but the number of residues that do not make direct polar contacts (yellow residues) is higher for the binding of RuBP compared with that of CABP (Figure 5). 3-PGA interacts with less residues and makes less polar contacts compared with RuBP (Figure 5), reinforcing the suggestion that 3-PGA binds more loosely to Rubisco catalytic sites than RuBP. The same response can be noticed for F6P and G6P, but the sequence of amino acids involved in interactions is slightly different to RuBP, suggesting that these two hexoses might regulate Rubisco activity differently to RuBP or 3-PGA.

## Discussion

This study investigated the direct effect of chloroplast metabolites on rice Rubisco activity *in vitro* to better understand how the enzyme may be regulated *in vivo* by metabolic changes. Rubisco activity often constrains photosynthesis, plant growth and yield. Its regulation is complex, involving interaction with various proteins and components of the chloroplast stromal environment [6]. The regulation of enzymes by metabolite levels is a fast and simple way to adjust metabolism, reducing energy costs and toxicity. Environmental conditions (e.g. heat and light intensity) can also modify cellular metabolic composition and activate or inactivate enzymes. The level of each metabolite in the chloroplast stroma under different growth stages and/or environmental conditions, and how this may be linked to the regulation of Rubisco activity, has yet to be thoroughly investigated. Our study shows that carbamylated rice Rubisco is directly down-regulated by CA1P (inhibitory sugar phosphate) and high concentrations of 3-PGA (product of its activity).

The synthetic Rubisco inhibitor, CABP binds tightly to carbamylated Rubisco catalytic sites, as it has a similar structure to the substrate RuBP, and in particular to the reaction intermediate 2-carboxy-3-keto-d-arabinitol-1,5-bisphosphate before the production of the two molecules of 3-PGA (Supplementary Table S1) [22]. CABP makes more polar contacts than RuBP and the other tested metabolites that strongly stabilize the binding between the ligand and protein [8,20]. The inhibitory mechanism is characterized by a competitive inhibition with a rapid equilibration, and a slow isomerization between enzyme/inhibitor, resulting in a non-exchangeable complex. The interaction with Mg^2+^ and L335 (loop 6 residue) is crucial for this tight-binding interaction [8,20]. In our study, CABP decreased the apparent *V*_max_ but not the apparent *K_m_* for both substrates, resembling an irreversible (or covalent) inhibition [23]. However, this mechanism would require a chemical reaction which has not been mentioned in the literature so far for Rubisco inhibition by sugar phosphates. Therefore, we believe that the mechanism involved is a strong fitting and polar interaction between CABP and Rubisco catalytic sites that is independent of substrate concentration [8,9,20]. This is consistent with the use of ^14^C-labelled CABP-binding assays as the most accurate method to quantify carbamylated Rubisco for more than 30 years [24,25].

To the best of our knowledge, the concentration of CA1P in rice leaves is unknown, but there is evidence that Rubisco is dark-inhibited in rice [26]. For this study we considered the concentration of CA1P found in wheat (also a *Poaceae* crop). This species has only 3.1% dark inhibition, much lower than some other species, but under light-acclimated conditions this still represents 0.17 µmol inhibitors/µmol Rubisco catalytic sites [7,27,28]. This suggests Rubisco dark regulation by CA1P plays an important role in wheat, which is supported by results using transgenic plants with altered CA1Pase levels [28], and the likely importance of CA1P in other grasses such as rice. CA1P binds less tightly than CABP possibly because its structure contains just one phosphate [29]. However, our results show that when CA1P is present in equal concentration to Rubisco catalytic sites (0.3 µM), Rubisco activity decreases by half. CA1P binds tightly to the Rubisco catalytic site, decreasing the apparent Rubisco maximum velocity ($V_{\max}^{\mathrm{RuBP}}$) and increasing the apparent Michaelis-Menten constant for RuBP ($K_{m}^{\mathrm{RuBP}}$) (Figure 4B,G). Naturally, it seems that CA1P and probably the other inhibitory sugar phosphates fit very well structurally in the Rubisco catalytic site, as they resemble its substrate RuBP, requiring the chaperone Rubisco activase to release them from the Rubisco catalytic sites, rendering a free carbamylated enzyme [7,8,30].

Metabolites produced downstream of Rubisco carboxylation or oxygenation reactions did not directly affect Rubisco activity, with the exception that 3-PGA seems to have a significant negative feedback regulation on Rubisco activity in rice. Chu and Bassham [13] reported that 0.5–2 mM 3-PGA slightly increased spinach Rubisco activity *in vitro*. In terms of metabolic regulation, this activity response would be unlikely to happen, so this could be an artefact of the assay. In our study, only the highest concentrations of 3-PGA (>4 mM) affected Rubisco activity. Depending on the species and growth conditions, this could be above-expected endogenous concentrations [31]. However, this kind of regulation could potentially happen in plants under stress conditions that promote the accumulation of carbohydrates in the leaves, either as a response to adjusting the osmotic potential or changes in the sink (non-photosynthetic tissues) activity [32,33]. Moreover, it will be important to consider this regulation both in the context of plants engineered for higher photosynthetic activity and in the predicted elevated atmospheric CO_2_, where Rubisco carboxylation activity in crops is expected to be higher than in the current atmospheric levels [34–37].

The accumulation of other carbohydrates (e.g. hexose phosphate, sucrose, and trehalose 6-phosphate) in the leaves has been associated with photosynthetic protein inhibition, including decreases in gene expression and activity of Rubisco [16,17,38]. Recently, a mechanism involving the down-regulation of chloroplast Mg^2+^ transporters (*OsMGT3*) via activation of circadian oscillator proteins (pseudo-response regulator) and superoxide $\left(O_{2}^{-}\right)$ signalling as a response to sugar accumulation was associated with photosynthesis inhibition in rice [39,40]. Spinach Rubisco activation was unaffected or only slightly affected by lower concentrations (<2 mM) of F6P and FBP, respectively [13]. Later, these authors observed that above 1 mM FBP competes with RuBP when added together to carbamylated Rubisco, indicating that this compound might regulate Rubisco activity in certain conditions [41]. In this study we observed a minor effect on Rubisco activity by high concentrations of F6P and G6P (6 mM), suggesting that these hexoses could interact with Rubisco in specific conditions, especially in conditions with limiting substrate concentrations (CO_2_ and/or RuBP). This regulation could become significant in conditions that decrease stomatal conductance and intercellular CO_2_ partial pressure (i.e. drought) or under low light, which limit RuBP regeneration. However, under normal *in vivo* conditions this is unlikely to occur as the concentration of these metabolites would typically be below 5 mM [42]. GAP and DHAP are structurally similar to 3-PGA (Supplementary Table S1), but the effect of these triose-phosphates on Rubisco activity was minimal or undetectable in the conditions tested. Moreover, these two metabolites can be quickly metabolized and do not accumulate under normal conditions [42], therefore would not be expected to play a major role in regulating Rubisco activity.

Other carbohydrates with no phosphate in their structure (ketoglutarate, malate and OAA) and animo acids (Gln, Glu and Gly, Supplementary Table S1), did not affect Rubisco activity in our study. This result corroborates Chastain and Ogren [14] who also observed no effect of 100 µM 2-oxoglutarate (similar in structure to OAA with one more methylene group) on spinach Rubisco activity in intact chloroplasts. In intact spinach chloroplasts, there was no effect on Rubisco activity when using 300 µM Glu and Gly [14]. However, high concentrations of Gly seem to chelate Mg^2+^ and be toxic to cyanobacteria [43]. Mg^2+^ concentration is important in stabilizing the carbamylated Rubisco conformation and supporting its activity. Our results suggest that *in vivo* concentrations (below 80 µM) of Gly do not affect Rubisco activity under saturating Mg^2+^ concentrations (20 mM). If Gly affects Rubisco activity under limiting Mg^2+^ concentrations (i.e. under Mg^2+^ deficiency or during dark-to-light or light-to-dark transitions where the stromal pH would be below 8) remains to be investigated.

The accumulation of photorespiratory compounds is considered toxic to plants and negatively correlated with photosynthesis, Rubisco activity and growth [19,44,45]. The activation of spinach Rubisco in intact chloroplasts was not affected by glycolate but was decreased by micromolar concentrations of glyoxylate (>500 µM), especially under low concentration of NaHCO_3_ (0.3 mM) [14,15]. The authors did not find any direct response of these metabolites on purified Rubisco or Rubisco activase activity. In fact, how photorespiratory compounds regulate photosynthesis is not entirely clear, although there is evidence that the accumulation of 2-PG, in 2-PG phosphatase loss-of-function mutants, decreases photosynthesis by inhibiting the activity of triose phosphate isomerase and sedoheptulose 1,7-bisphosphatase [46,47]. In our study, we did not detect any interaction between 2-PG, glycolate, or glyoxylate with carbamylated rice Rubisco in both saturating and limiting concentrations of substrates. Therefore, it seems that photorespiratory compounds do not interact with Rubisco and that down-regulation of photosynthesis and Rubisco activity *in vivo* is through indirect mechanisms.

Rubisco synthesis and activity are highly regulated *in vivo*. It is a complex and key enzyme for photosynthesis and plant growth [1,6]. However, the carbamylated Rubisco conformation is relatively selective and regulated by a few specific chloroplast metabolites. Our results demonstrate that rice carbamylated Rubisco activity is strongly inhibited by CA1P and moderately inhibited by relatively high concentrations of 3-PGA. These two metabolites compete with RuBP by binding tightly and loosely, respectively, to Rubisco catalytic sites. The effects on the $V_{\max}^{CO_{2}}$ and $K_{m}^{CO_{2}}$ seem to be a consequence of the competition of these metabolites with RuBP rather than an impairment of CO_2_ binding. For the purpose of this study, we focused on the inhibition of RuBP carboxylation at atmospheric oxygen concentration (21%), rather than the inhibitory effect of O_2_ competition with CO_2_ for reaction with RuBP. During the carboxylation reaction, RuBP binds to a carbamylated catalytic site of Rubisco, it then suffers a deprotonation at C3 leading to an unstable enediolate intermediate, which is directly attacked by CO_2_ at C2, generating a six-carbon intermediate, and this is later hydrolytically cleaved into two molecules of 3-PGA [1]. Therefore, if the catalytic site is blocked by compounds, such as CA1P or 3-PGA, the number of active functional catalytic sites decreases, lowering apparent $V_{\max}^{CO_{2}}$ and, consequently, reducing the apparent $K_{m}^{CO_{2}}$. This indicates that the apparent $K_{m}^{CO_{2}}$ decreased as a consequence of the lower availability of free catalytic sites instead of changes in the affinity of Rubisco for CO_2_.

It is also conceivable that at certain concentrations of substrate CO_2_, CA1P and 3-PGA might have an apparent effect of Rubisco activation illustrated by the reaction velocity in the presence of the effector $\left(v_{0}^{\prime }\right)$ being similar to the velocity of the control (*v*_0_), decreasing the apparent $K_{m}^{CO_{2}}$ [48]. Considering the whole L_8_S_8_ complex, Rubisco has eight catalytic sites, with two within each of the large subunit antiparallel dimers. Thus, it may be that when one site is inhibited, the conformation of the complex might change and affect the affinity of the other catalytic sites for the substrates. These results also suggest that the binding of CA1P and 3-PGA to Rubisco catalytic sites is stronger under limiting CO_2_ concentration than under saturating CO_2_ and limiting RuBP concentrations. CA1P decreased the apparent *V*_max_ for both substrates suggesting that it was locked in the catalytic site decreasing the apparent $K_{m}^{CO_{2}}$, but as it increased the apparent $K_{m}^{\mathrm{RuBP}}$, this response suggests that high concentrations of RuBP would replace some CA1P, as CA1P binds less tightly than CABP. Conversely, 3-PGA decreased the apparent $V_{\max}^{CO_{2}}$ and did not affect the apparent $V_{\max}^{\mathrm{RuBP}}$, suggesting a tighter binding under limiting CO_2_ concentration than under limiting RuBP concentration. The changes in the Rubisco kinetics for RuBP effected by 3-PGA are representative of classic competitive inhibition, while the effects of inhibition by CABP and CA1P would be representative of mixed or noncompetitive. This is an oversimplification as tight-biding inhibitors require more laborious methods for estimation of *K_i_*, however the apparent *K_i_* estimates support the binding strengths for CABP (very tight), CA1P (tight) and 3-PGA (loosely) to Rubisco catalytic sites.

The results also highlight the relevance of Rubisco activity regulation by CA1P in rice, a trait known to vary significantly across different plant groupings [7]. Further investigations of CA1P metabolism, abundance during the diel cycle, and impact on Rubisco activity *in vivo* would improve our understanding of Rubisco regulation in rice. A better understanding of how chloroplast metabolites affect Rubisco activation or activity under different conditions (e.g. different temperatures, pH or Mg^2+^ concentration) by interacting with catalytic or allosteric sites could be achieved by combining biochemical analyses with protein structural crystallography and molecular docking approaches.

## Methods

### Rice Rubisco purification

Rice (*Oryza sativa* L., cv. Nipponbare) plants were cultivated in a semicontrolled glasshouse with a 28/23°C day/night temperature, photoperiod of 13 h and 30% relative humidity. During the day, supplemental lighting was used to maintain a minimum light level of 600 µmol m^−2^ s^−1^. Seeds were incubated in distilled water for 48 h at 37°C and sowed in 5-L pots containing Norfolk Topsoil (Bayley's of Norfolk Ltd, U.K.). Plants were kept in continuous water saturation conditions. Rubisco was extracted from young and healthy leaves harvested ∼3 weeks after sowing. The harvesting was performed 3–4 h after the beginning of the photoperiod. Rubisco purification was performed as described by Amaral et al. [49] and quantified by the [^14^C]CABP binding assay [25]. The enzyme was activated by incubation with 0.1 M Bicine, pH 8.0, containing 20 mM MgCl_2_ and 110 µM CO_2_ (10 mM NaHCO_3_) at 37°C for 40 min prior quantification and assays. SDS and Native gels (Mini-PROTEAN TGX 10% polyacrylamide gel, Bio-Rad, U.K.) were performed to check enzyme purity according to the manufacturer's protocol (Supplementary Figure S6).

### Chloroplast metabolites dose-response curves

To understand how different chloroplast metabolites affect the activity of carbamylated Rubisco *in vitro*, metabolite dose-dependent response curve assays were performed. Rubisco *in vitro* activity was determined immediately upon enzyme activation, via incorporation of ^14^CO_2_ into stable sugars as detailed in [50] with minor changes. Activated Rubisco was incubated at 30°C for 5 min in a reaction mixture containing assay buffer (100 mM Bicine-NaOH, pH 8.2, 20 mM MgCl_2_, 110 µM CO_2_ (10 mM NaH^14^CO_3_, 9.25 kBq µmol^−1^), 2 mM KH_2_PO_4_), and 25 µl of each individual metabolite concentration. The range of metabolite concentrations tested included concentrations expected *in vivo* at normal conditions, as well as lower and higher physiological concentrations ([31,42,46,51–56] Supplementary Table S1). Afterwards, Rubisco *in vitro* activity was started by adding 600 µM RuBP and quenched after 30 s by adding 10 M formic acid. Zero metabolite (RO water) was used as a control and Rubisco concentration in the assay was ∼15.64 µg/ml. These assays (assay 1) were performed under saturating substrates (RuBP and CO_2_) and atmospheric O_2_ (21%) concentrations.

The metabolites and concentrations tested are listed in the Supplementary Table S1, including the synthetic (CABP) and natural (CA1P) inhibitory sugar phosphates, photorespiratory metabolites (2-PG, glycolate and glyoxylate), carbohydrates (3-PGA, glyceraldehyde-3-phosphate (GAP), DHAP, fructose-6-phosphate (F6P), glucose-6-phosphate (G6P), ketoglutarate, malate and oxaloacetate (OAA)) and amino acids (glutamine (Gln), glutamate (Glu) and glycine (Gly)). CABP and CA1P were synthesised in the lab as described in Kingston-Smith et al. [57], and all the other metabolites were purchased from Sigma–Aldrich/Merck with high optical purity (≥93%). CABP is a synthetic compound which resembles RuBP (Rubisco substrate) and has a high affinity to carbamylated conformation Rubisco catalytic sites, so it was used for comparison. The maximum concentration of CABP tested corresponds to the concentration of Rubisco catalytic sites, while the maximum metabolite concentration/Rubisco catalytic sites was much higher for all the other metabolites. These results were expressed as % of control (zero metabolite) samples (where control is 100%).

### Rubisco maximum velocity and Michaelis-Menten constant for CO_2_ and RuBP

After identifying which metabolites affected the carbamylated Rubisco activity, Rubisco activity assays were performed at different substrates concentrations and two concentrations of the selected metabolites to estimate the apparent (at atmospheric O_2_ concentration) maximum velocity (*V*_max_) and the substrate affinity (Michaelis-Menten constant — *K_m_*) for CO_2_ and RuBP. Water was used as control and for each metabolite an intermediate and the highest concentration were used, with the exceptions of CABP for which the maximum concentration was 0.2 µM (as at the maximum concentration 0.3 µM the activity was very low) and for 3-PGA we used 8 and 12 mM (as these were the concentrations that affected Rubisco activity under saturating substrate concentrations). Basic assay buffer (100 mM Bicine-NaOH, pH 8.2, 20 mM MgCl_2_ and 2 mM KH_2_PO_4_) was sparged with compressed air which passed through soda lime and 1 M NaOH to remove CO_2_. For the CO_2_ curve, Rubisco activity was measured at 4, 16, 36, 68 and 100 µM CO_2_ and 600 µM RuBP in sealed vials. For the RuBP curve, Rubisco activity was measured at saturating CO_2_ (110 µM) and 10, 50, 150, 300 and 600 µM RuBP in open vials. The incubation with metabolite and activity were performed as described above. *V*_max_ and *K_m_* were calculated using the Michaelis-Menten equation [58]. The effect of all other metabolites that did not affect Rubisco activity under saturating substrates concentration in the metabolite dose-response curves was also investigated under limiting CO_2_ and RuBP concentrations. Rubisco activity was quantified in the presence of the highest concentration of each metabolite and low CO_2_ (8 µM) or low RuBP (50 µM). The incubation and activity were performed as described above and the activity was expressed as % of control samples (where control is 100%).

Given the constraints associated with two substrate Rubisco activity and its interaction with tight binding inhibitions, we used a simplistic approximation to estimate apparent inhibitor constants $\left(K_{i}^{\mathrm{app}}\right)$ based on the Michaelis-Menten equations [59] (eqn 1) and on the ‘quotient velocity plot’ [60] (eqn 2). Where [*I*] is the inhibitor concentration and α was estimated according to the inhibition type:

**Table:** 

Inhibition Type	eqn 1 [59]	eqn 2 [60]
Noncompetitive-like (CA1P, CABP)	$K_{i}^{\mathrm{app}}$ = [*I*]/(α-1), *α* = *V*_max_/$V_{\max}^{\prime }$	(*V*_max_-*v*)/*v* = *K_m_*/[*S*] + (*K_m_*/[*S*] + 1) [*I*]/$K_{i}^{\mathrm{app}}$
Competitive-like (3-PGA)	$K_{i}^{\mathrm{app}}$ = [*I*]/(α-1), *α* = $K_{m}^{\prime }$/*K_m_*	(*V*_max_-*v*)/*v* = (1+ [*I*]/$K_{i}^{\mathrm{app}}$) *K_m_*/[*S*]

*K_m_* and *V*_max_ represent the values in the absence of inhibitor and $K_{m}^{\prime }$ and $V_{\max}^{\prime }$ represent the apparent values in the presence of inhibitor, *v* represents the velocity at the maximum concentration of substrate [*S*] in the presence of inhibitor.

### Molecular docking analysis

The docking analysis was performed by using the software GOLD in Herms platform (version 2023.3.0, CCDC, Cambridge, England). The crystal structure of carbamylated rice Rubisco (in a closed conformation) and metabolites were extracted from Protein Data Bank (PDB — Rubisco and CABP ID: 1WDD, RuBP ID: 9RUB, 3-PGA ID: 1AA1, F6P ID: 2OWZ, G6P ID: 7ZHV). For the docking analysis, waters were removed, and the docking was performed in the chain A of the 1WDD protein, using the CABP site (CAP1001) as the binding site and CABP as a reference ligand. The configuration template used was chemscore_kinase, the fitness function was CHEMPLP and the other parameters were set as default. The docking presenting the lowest PLP.PLP score was selected as best fitting, the parameters are presented in Table 1 and the structures were analysed in The PyMOL Molecular Graphics System (version 1.3 Schrödinger, LLC). The amino acid residues at a distance lower or equal to 4 Å from each ligand are presented in Figure 5A.

### Statistical analysis

Data was analysed using R 4.3.1 and RStudio 2023.06.1, and graphs were prepared using the *ggplot2* package [61]. Outliers were detected using the *rstatix* package, where the 1.5 times the interquartile range (1.5 IQR) below the first quartile or 1.5 IQR above the third quartile are considered outliers. Linear and polynomial models were created using the *stats* and *mgvc* packages [62] and the nonlinear-least-squares was performed using the *nls* function. The best-fit model was selected based on the lower Akaike information criterion [63]. *V*_max_ and *K_m_* were estimated according to the Michaelis-Menten equation using the R package *drc* and the function *drm* and the model was fitted using the *nls* function. Box plots show medians and the first and third quartiles (25th and 75th percentiles), and whiskers extend from the hinge to the largest or smallest value. Symbols represent individual data points (analytical replicate). Assays were carried out using technical replicates of the same Rubisco preparation, with a small number of assays repeated with a second preparation to corroborate the findings. The large proportion of data presented reflects three or more technical replicates (means and standard errors of the mean), with a minimal number of metabolite concentrations including two replicates that were consistent among them (as noted in figure captions). One-way analysis of variance followed by Tukey's *post hoc* test was used to test the significance of differences between metabolite concentrations at 5% of probability (*P* < 0.05). *t*-tests were used to assess pairwise differences between specific metabolite concentrations and the control in Supplementary Figures S4 and S5.

## Data Availability

The original data presented in the study are included in the article and supplementary material; and publicly available via the FAIRDOMHub repository: https://fairdomhub.org/data_files/7410.

## Abbreviations

2-PG: 2-phosphoglycolate
3-PGA: 3-phosphoglyceric acid
CABP: 2-carboxy-D-arabinitol-1,5-bisphosphate
CA1P: 2-carboxy-D-arabinitol 1-phosphate
DHAP: dihydroxyacetone phosphate
F6P: fructose 6-phosphate
FBP: fructose-1,6-bisphosphate
GAP: glyceraldehyde-3-phosphate
G6P: glucose 6-phospahte
Gln: glutamine
Glu: glutamate
Gly: glycine
Ki app: apparent inhibitor constant
Km: apparent Michaelis-Menten constant
OAA: oxaloacetic acid
RuBP: ribulose-1,5-bisphosphate
Vmax: apparent maximum velocity
