# Supplementary material for: Regulation of Rubisco activity by interaction with chloroplast metabolites
Source: Biochem J. 2024 Aug 5;481(15):1043–56. doi: 10.1042/BCJ20240209 (PMC11346435; doi:10.1042/BCJ20240209)
Supplement: Supplementary Material [file BCJ-481-1043-s1.pdf]

Supplementary Material - Figures

Regulation of Rubisco activity by interaction with chloroplast metabolites

Ana KM Lobo, Douglas J Orr, Elizabete Carmo-Silva

Lancaster Environment Centre, Lancaster University, Lancaster, LA1 4YQ, UK

Correspondence: Elizabete Carmo-Silva (e.carmosilva@lancaster.ac.uk) or Ana KM Lobo (a.moreiralobo@lancaster.ac.uk)

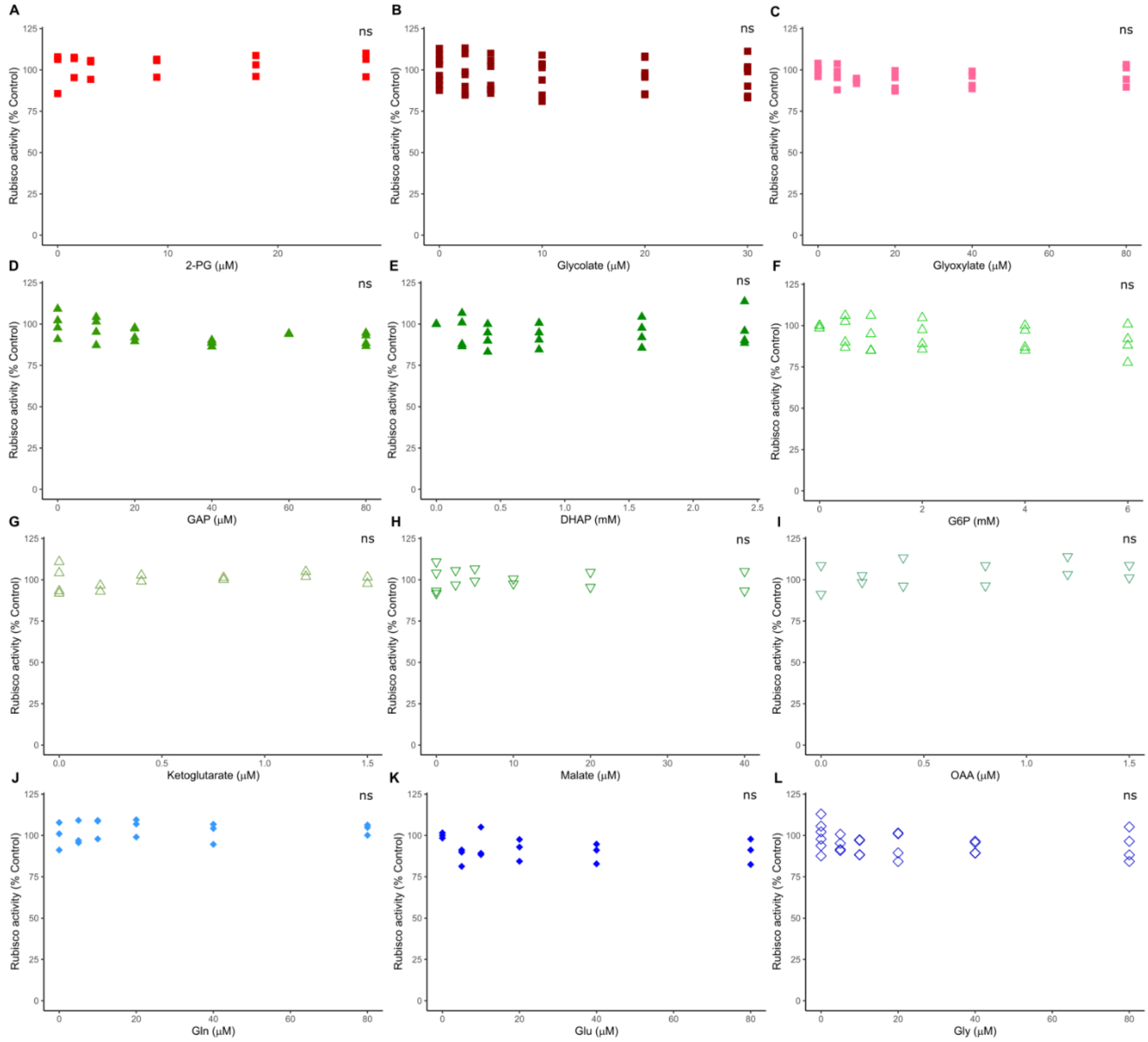

Figure S1: Effect of chloroplast metabolites dose-response curve on rice Rubisco activity. 2-Phosphoglycolate (A), Glycolate (B), Glyoxylate (C), Glyceraldhyde-3-phosphate (D), Dihydroxyacetone phosphate (E), Glucose 6-phosphate (F), Ketoglutarate (G), Malate (H), Oxaloacetic acid (I), Glutamine (J), Glutamate (K) and Glycine (L). Symbols represent individual samples (n = 3-7 technical replicates except for malate, oxaloacetate and ketoglutarate that had 2 technical replicates). Lines were omitted as they were not significant (ns).

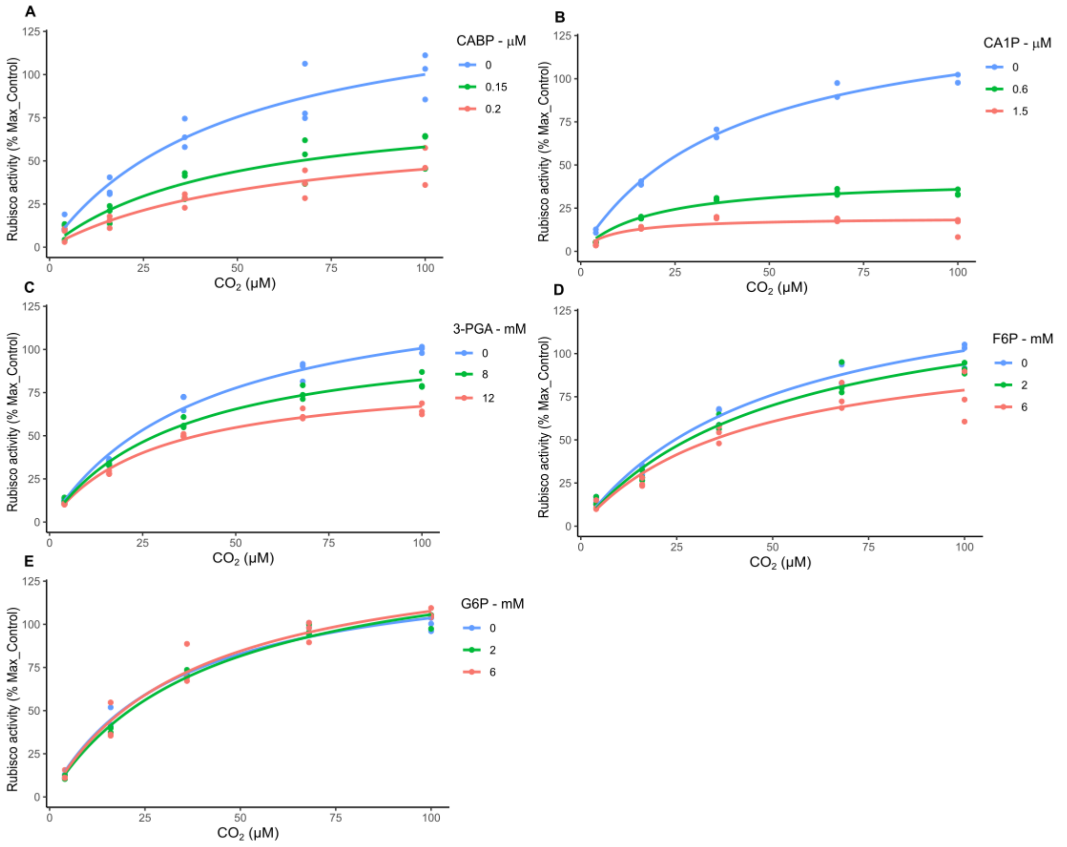

Figure S2: CO_2 dose-response curve of Rubisco activity in presence of different concentrations of chloroplast metabolites: 2-carboxy-D-arabinitol-1,5-bisphosphate (A), 2-carboxy-D-arabinitol 1-phosphate (B), 3-Phosphoglycerate (C), Fructose 6-phosphate (D) and Glucose 6-phospahte (E). The activity is expressed as % of the maximum activity of the control (0 metabolite) at 100 µM CO_2 (average = 1.78 ± 0.11 µmol mg^{-1} protein min^{-1}). Symbols represent individual samples (n = 3 technical replicates; except for 0 µM CA1P with 2 technical replicates).

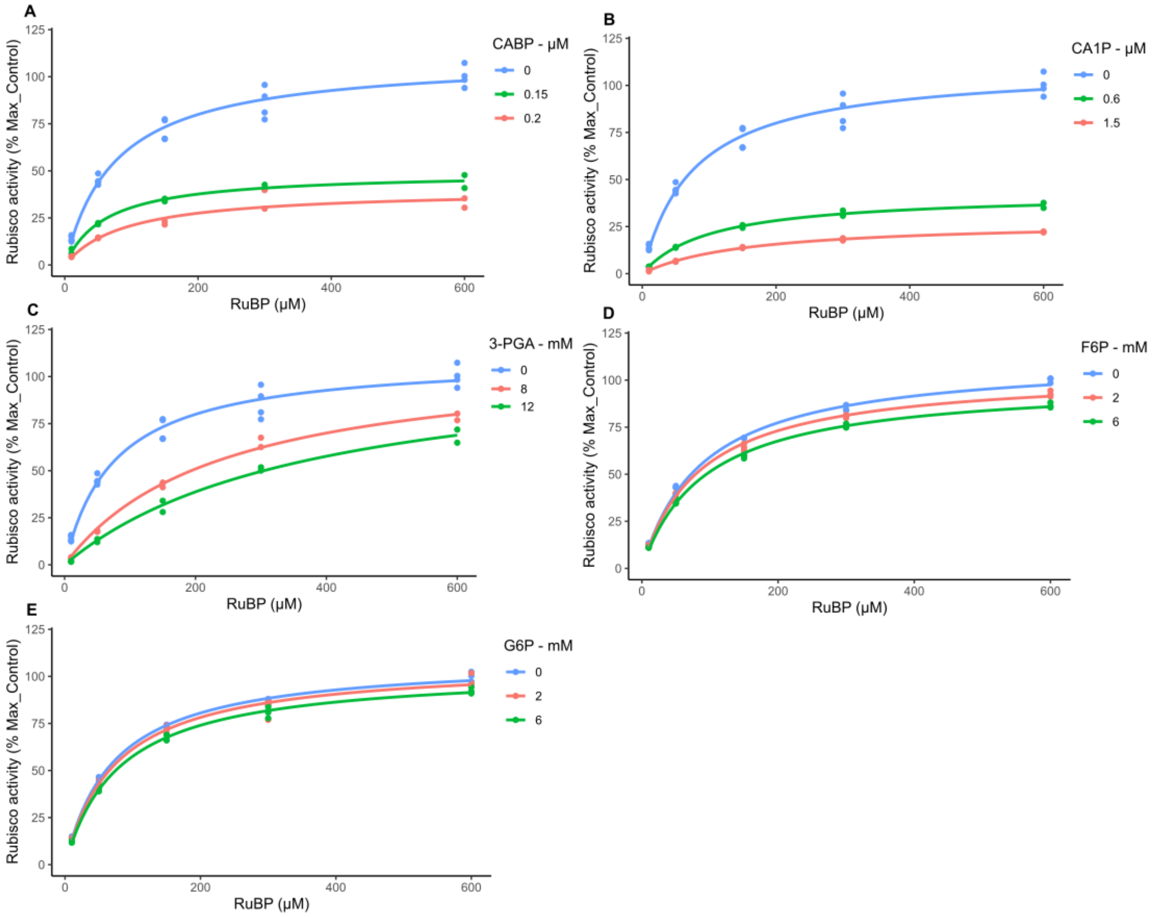

Figure S3: RuBP dose-response curve on Rubisco activity in the presence of different concentrations of chloroplast metabolites: 2-carboxy-D-arabinitol-1,5-bisphosphate (A), 2-carboxy-D-arabinitol 1-phosphate (B), 3-Phosphoglycerate (C), Fructose 6-phosphate (D) and Glucose 6-phospahte (E). The activity is expressed as % of the maximum activity of the control (0 metabolite) at 600 µM RuBP (average = 1.44 ± 0.14 µmol mg^{-1} protein min^{-1}). Symbols represent individual samples (n = 3-4 technical replicates).

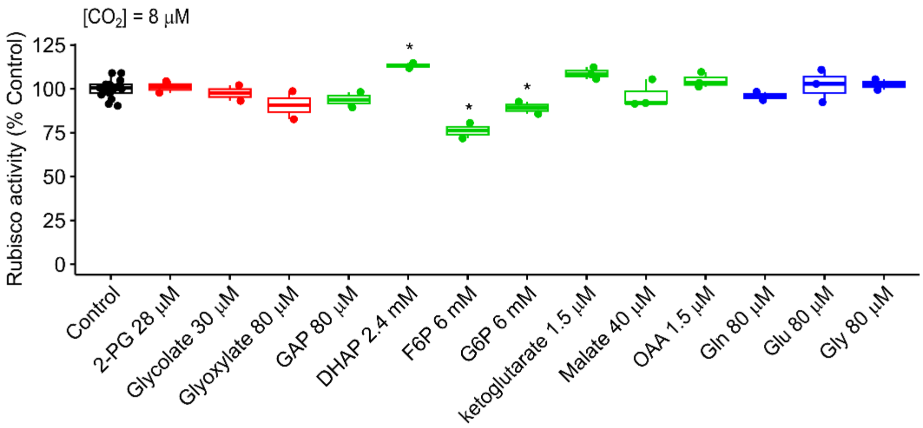

Figure S4: Rubisco activity in the presence of the highest concentration of chloroplast metabolite and low concentration of CO_2 (8 µM). Boxes represent the median and first and third quartiles, whiskers represent the range, symbols represent individual samples (n = 2-3 technical replicates). Asterisk represents significant differences between the control and metabolite at 5% level according to *t*-test ($p \leq 0.05$). Control average = 0.49 ± 0.03 µmol mg^{-1} protein min^{-1}.

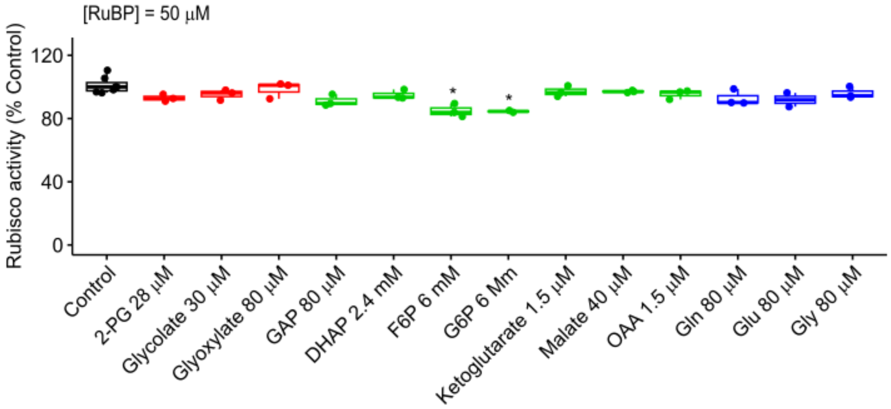

Figure S5: Rubisco activity in the presence of the highest concentration of chloroplast metabolite and low concentration of RuBP (50 µM). Boxes represent the median and first and third quartiles, whiskers represent the range, symbols represent individual samples (n = 3 technical replicates except for G6P and Glu with 2 technical replicates). Asterisk represents significant differences between the control and metabolite at 5% level according to *t*-test ($p \leq 0.05$). Control average = 0.65 ± 0.07 µmol mg^{-1} protein min^{-1}.

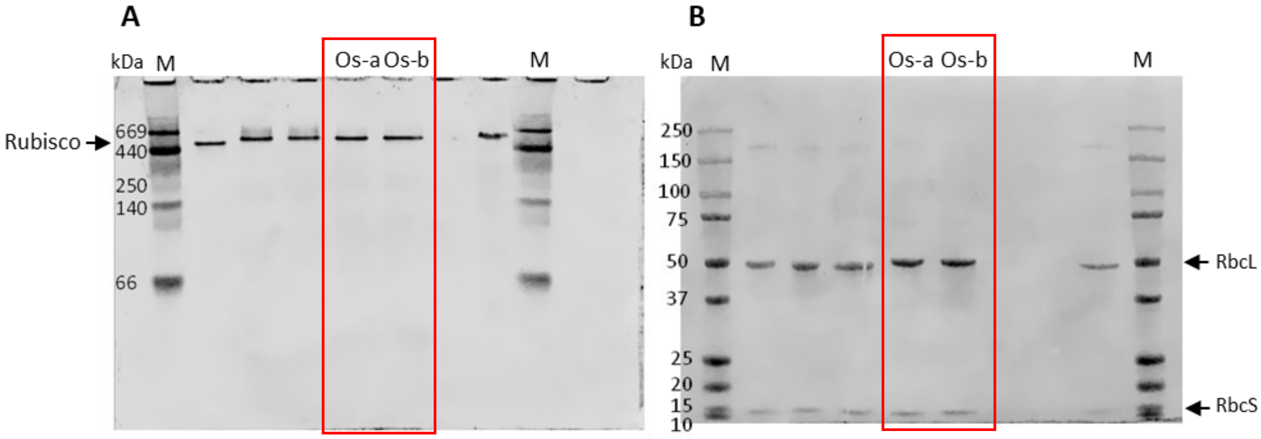

Figure S6: Purified rice Rubisco (Os-a and Os-b). Native (A) and SDS denaturing (B) polyacrylamide gel electrophoresis stained with Coomassie Blue. Each gel lane was loaded with 1 µg of total soluble protein. M = molecular marker. The red box denotes the protein preparations used in this study.

Supplementary Material - Tables

Regulation of Rubisco activity by interaction with chloroplast metabolites

Ana KM Lobo, Douglas J Orr, Elizabete Carmo-Silva

Lancaster Environment Centre, Lancaster University, Lancaster, LA1 4YQ, UK

Correspondence: Elizabete Carmo-Silva (e.carmosilva@lancaster.ac.uk) or Ana KM Lobo (a.moreiralobo@lancaster.ac.uk)

Table S1: Concentrations of metabolites in plant chloroplast used in activity assays with purified rice Rubisco. Water (0 metabolite) was always used as control; the second and third intermediate values correspond to the concentration range likely to be physiologically relevant for regulating the activity of Rubisco *in vivo*. na = not applicable. [1]Structure source: https://pubchem.ncbi.nlm.nih.gov/. Source: metabolites were synthesised in the lab as described in the methods (Synt.) or purchased from Sigma-Aldrich/Merk (Sigma as indicated with product number).

Metabolite name and structure[1]	Metabolite abbreviation	Concentrations used	Source	Reference for physiological concentration in chloroplasts
Ribulose-1,5-bisphosphate 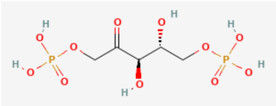	RuBP	Rubisco substrate used under limiting (10, 50, 150, 300 µM) and saturating (600 µM) concentrations	Synt.	Schimkat et al., 1990 [31]
2-carboxy-D-arabinitol-1,5-bisphosphate 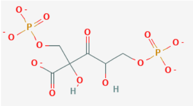	CABP	0.05, 0.1, 0.15, 0.2, 0.3 µM	Synt.	Synthetic, not present *in vivo*. Maximum concentration corresponding to the concentration of Rubisco catalytic sites used in the assays.
2-carboxy-D-arabinitol 1-phosphate 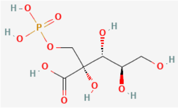	CA1P	0.15, 0.3, 0.6, 1, 1.5 µM	Synt.	Moore et al., 1991 [51]
2-Phosphoglycolate 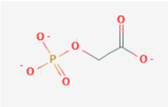	2-PG	1.5, 3, 9, 18, 28 µM	Sigma: 72764	Flügel et al., 2017 [46]
Glycolate 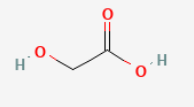	na	2.5, 5, 10, 20, 30 µM	Sigma: 124737	Xu et al., 2009 [52]
Glyoxylate 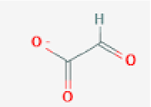	na	5, 10, 20, 40, 80 µM	Sigma: G10601	Xu et al., 2009 [52]
3-Phosphoglycerate 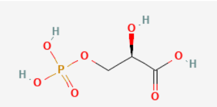	3-PGA	1, 2, 4, 8, 12 mM	Sigma: P8877	Zhu et al., 2007 [53]
Glyceraldehyde-3-phosphate 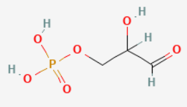	GAP	10, 20, 40, 60, 80 µM	Sigma: 69312	Zhu et al., 2007 [53]

Dihydroxyacetone phosphate 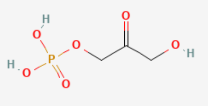	DHAP	0.2, 0.4, 0.8, 1.6, 2.4 mM	Sigma: 51269	Zhu et al., 2007 [53]
Fructose 6-phosphate 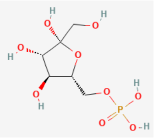	F6P	0.5, 1, 2, 4, 6 mM	Sigma: F3627	Bassham & Krause, 1969 [42]
Glucose 6-phosphate 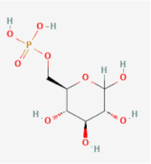	G6P	0.5, 1, 2, 4, 6 mM	Sigma: G7250	Bassham & Krause, 1969 [42]
Ketoglutarate 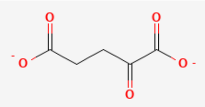	na	0.2, 0.4, 0.8, 1.2, 1.5 µM	Sigma: 75890	Szecowka et al., 2013 [51]
Malate 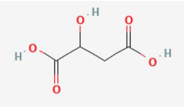	na	2.5, 5, 10, 20, 40 µM	Sigma: 02288	Szecowka et al., 2013 [54]
Oxaloacetic acid 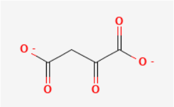	OAA	0.2, 0.4, 0.8, 1.2, 1.5 µM	Sigma: O4126	Isherwood & Niavis, 1956 [55]
Glutamine 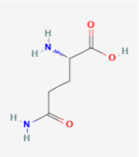	Gln	5, 10, 20, 40, 80 µM	Sigma: G3126	Dellero et al., 2015 [53]
Glutamate 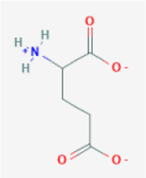	Glu	5, 10, 20, 40, 80 µM	Sigma: G1251	Dellero et al., 2015 [56]
Glycine 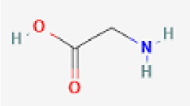	Gly	5, 10, 20, 40, 80 µM	Sigma: G8898	Dellero et al., 2015 [56]

Table S2: Model fitting to the dose-response of chloroplast metabolite on rice Rubisco activity. The model providing the best fit to the data is highlighted in bold and was selected according to the lowest AIC score (Akaike information criterion) estimated according to Akaike (1974) using the AIC function in R. Models were applied to the full dataset shown in Figure 2 and Supplementary Figure 1. nls = nonlinear least squares. *The nls was selected as a better representation of nonlinear fitting and the AIC is close to the minimal. R^2 and p-value were extracted from the linear regression and represent how the data fits in the model and the significance ($p < 0.05$ in bold), respectively.

Metabolite	Model	AIC score	R^2	p-value
CABP	Linear	155.88	0.95	**<0.001**
	2nd order polynomial	134.86		
	**nls***	**135.51**		
CA1P	Linear	199.21	0.77	**<0.001**
	2nd order polynomial	170.21		
	**nls**	**129.68**		
3-PGA	**Linear**	**158.22**	**0.69**	**<0.001**
	2nd order polynomial	160.17		
2-PG	Linear	124.56	0.02	0.59
	2nd order polynomial	126.56		
Glycolate	Linear	320.66	0.01	0.55
	2nd order polynomial	322.50		
Glyoxylate	Linear	146.69	0.00	0.84
	2nd order polynomial	144.23		
GAP	Linear	146.63	0.23	0.09
	2nd order polynomial	145.29		
DHAP	Linear	158.14	0.00	0.78
	2nd order polynomial	159.00		
F6P	**Linear**	**119.93**	**0.33**	**0.01**
	2nd order polynomial	121.38		
G6P	Linear	163.95	0.11	0.13
	2nd order polynomial	165.82		
Ketoglutarate	Linear	90.47	0.02	0.51
	2nd order polynomial	92.40		
Malate	Linear	94.25	0.00	0.75
	2nd order polynomial	96.25		
OAA	Linear	85.08	0.11	0.30
	2nd order polynomial	86.92		
Gln	Linear	119.08	0.01	0.65
	2nd order polynomial	120.82		
Glu	Linear	124.65	0.06	0.34
	2nd order polynomial	125.60		
Gly	Linear	179.75	0.04	0.31
	2nd order polynomial	180.22		

Table S3: Apparent inhibitor constants (Ki^{app}) of RuBP carboxylation for CABP (2-carboxy-D-arabinitol-1,5-bisphosphate), CA1P (2-carboxy-D-arabinitol 1-phosphate) and 3-PGA (3-Phosphoglycerate). Ki was estimated according to the type of inhibition and using Michaelis-Menten equations (Eq. 1) or the "quotient velocity plot" (Eq. 2) as detailed in the methods. For a more accurate and laborious method to determine the Ki for Rubisco inhibitors, please see Pearce and Andrews (2003 J Biol Chem 278: 32526-32536) [8].

Metabolite	Inhibition Type	Ki^{app} (Eq. 1)	Ki^{app} (Eq. 2)
CABP	Noncompetitive-like	0.12 ± 0.02 µM	0.10 ± 0.02 µM
CA1P	Noncompetitive-like	0.44 ± 0.08 µM	0.44 ± 0.01 µM
3-PGA	Competitive-like	3.36 ± 0.71 mM	3.21 ± 0.77 mM
